# Hyaluronan's Role in Fibrosis: A Pathogenic Factor or a Passive Player?

**DOI:** 10.1155/2015/790203

**Published:** 2015-10-25

**Authors:** Sami Albeiroti, Artin Soroosh, Carol A. de la Motte

**Affiliations:** Department of Pathobiology, Cleveland Clinic Lerner Research Institute, 9500 Euclid Avenue, Cleveland, OH 44195, USA

## Abstract

Fibrosis is a debilitating condition that can lead to impairment of the affected organ's function. Excessive deposition of extracellular matrix (ECM) molecules is characteristic of most fibrotic tissues. Fibroblasts activated by cytokines or growth factors differentiate into myofibroblasts that drive fibrosis by depositing ECM molecules, such as collagen, fibronectin, and connective tissue growth factor. Transforming growth factor-*β* (TGF-*β*) is one of the major profibrotic cytokines which promotes fibrosis by signaling abnormal ECM regulation. Hyaluronan (HA) is a major ECM glycosaminoglycan that is regulated by TGF-*β* and whose role in fibrosis is emerging. Aside from its role as a hydrating, space filling polymer, HA regulates different cellular functions and is known to have a role in wound healing and inflammation. Importantly, HA deposition is increased in multiple fibrotic diseases. In this review we highlight studies that link HA to fibrosis and discuss what is known about the role of HA, its receptors, and its anabolic and catabolic enzymes in different fibrotic diseases.

## 1. Introduction

*(1) Extracellular Matrix Remodeling in Fibrosis.* A significant volume of living tissues is occupied by extracellular space that is filled with the extracellular matrix (ECM). Molecules of the ECM, fibrous proteins, and glycosaminoglycans (GAGs) are organized at the surface of the cells that produce them and form a network between cells [1]. Aside from its classical role in structure and support, the ECM has a major role in cell regulation, migration, proliferation, and survival. While ECM remodeling is very important in the normal development, many studies have also linked the remodeling of ECM to disease pathogenesis and, notably, to fibrosis [2]. Fibrosis, the formation of scar tissue that results from excessive aberrant wound healing, is characterized by a significant increase in ECM deposition. Inflammation is known to be a major contributing factor to fibrosis. Whereas mild inflammation ends in the restoration of normal tissue architecture, severe and chronic inflammation result in tissues losing their ability to heal. This promotes a fibrogenic repair response, the excessive accumulation of ECM, and the formation of abnormal tissue architecture. Subsequently, sustained fibrosis leads to impaired organ function [3, 4].

Fibrosis is driven primarily by myofibroblasts. Fibroblasts are normally classified as major ECM-producing cells in the human body. When activated, fibroblasts differentiate into a myofibroblastic cell type that expresses alpha smooth muscle actin (*α*-SMA) and overexpress collagen and fibronectin [5, 6]. Fibroblast activation, proliferation, differentiation, and migration initiate the fibrotic process. Fibroblasts can be activated by many inflammatory mediators, among which are tumor necrosis factor (TNF-*α*), platelet-derived growth factor (PDGF), interleukin-33, interleukin-13, and transforming growth factor beta (TGF-*β*), the well-studied and major mediator of fibrosis that acts through SMAD-dependent and SMAD-independent signaling pathways [4, 7–10].

After tissue injury, TGF-*β* levels are significantly increased, which aids in recruiting immune cells, like neutrophils and macrophages, and activating fibroblasts, which release further TGF-*β*. TGF-*β* mediates the deposition of many ECM proteins, including collagen and fibronectin, and matricellular proteins (ECM-bound proteins that have regulatory and signaling roles but not structural roles) such as connective tissue growth factor (CTGF) [11]. Through the action of SMAD proteins, TGF-*β* promotes collagen deposition both by enhancing the expression of different types of collagen genes and by mediating the overexpression of collagenase inhibitors [12]. TGF-*β* also promotes the expression and deposition of another major ECM protein, fibronectin, specifically the extra domain A- (EDA-) containing fibronectin. EDA-fibronectin has a major role in myofibroblast differentiation and wound healing and its expression is upregulated during tissue repair and scar formation [13]. Interestingly, Bhattacharyya et al. have recently shown that EDA-fibronectin is significantly increased in mice with bleomycin-induced cutaneous fibrosis and, importantly, is a ligand for toll-like receptor- (TLR-) 4. The group also showed that,* in vitro*, EDA-fibronectin treatment stimulated myofibroblast differentiation and collagen production and the effect was blocked by blocking TLR4 signaling [14]. A third major mediator of tissue remodeling and fibrosis that is stimulated by TGF-*β* is CTGF. CTGF is a matricellular protein that is known to interact with different cytokines and cellular receptors as well as with other ECM proteins and is expressed only during the process of wound repair. Not only is CTGF overexpressed during scar formation, but it is also required for persistent TGF-*β*-driven fibrosis, as seen in a mouse fibrosis model where CTGF inhibition has been shown to prevent and reverse fibrosis [15].

In addition to the abovementioned ECM proteins that are involved in fibrogenesis, proteoglycans and GAGs have also been shown to be involved in this process. Evidence shows that the deposition of versican, a chondroitin sulfate-containing proteoglycan, is significantly increased in the ECM of lung lesions from patients with idiopathic pulmonary fibrosis [16]. In bleomycin-induced pulmonary fibrosis in rats, the increased levels of TGF-*β* associated with a significant increase in biglycan mRNA and a decrease in decorin mRNA, both of which are classified as small leucine-rich proteoglycans [17]. Another report has shown, in a rat model of a bleomycin-induced pulmonary fibrosis, that levels of versican, heparin sulfate, and fibromodulin are increased in fibrotic lungs [18]. Importantly, the same group has shown that alterations in proteoglycan and GAGs are associated with alterations in the viscoelastic properties of lung parenchymal tissues early in the fibrotic response [19]. This change in the viscoelastic properties of the lung during fibrosis could partially explain the impaired function of the organ. Additionally, alterations in the expression of enzymes implicated in synthesis and sulfation of GAGs have been reported in fibrosis. Fibroblasts derived from fibrotic lungs expressed increased mRNA levels of xylosyltransferase-I and chondroitin-4-sulfotransferase-I compared to their nonfibrotic counterparts. However, expression of these two enzymes was increased in the nonfibrotic lung fibroblasts in response to TGF-*β* stimulation via p38 mitogen-activated protein kinase (MAPK) and TGF-*β* type-1 receptor/activin receptor-like kinase 5 pathways [20]. Collectively, published data suggest that remodeling of ECM during fibrosis is a regulated process that involves the activity of anabolic enzymes and catabolic enzymes. An additional major ECM molecule that has an underappreciated but rapidly emerging role in the fibrotic process is hyaluronan (HA).

*(2) Hyaluronan.* HA is an ubiquitous, nonsulfated, unbranched GAG and the largest polysaccharide produced in vertebrates. It is the only GAG that does not have a core protein component. The HA chain is made up of repeating disaccharides; each disaccharide is composed of D-*N*-acetylglucosamine and D-glucuronic acid linked by alternating *β*-(1,4) and *β*-(1,3) glycosidic bonds [21]. Three mammalian HA synthase (HAS) enzymes have been identified: HAS1, HAS2, and HAS3; their structure is well-conserved among various mammalian species. HA is synthesized uniquely and unlike other GAGs that are synthesized in the Golgi apparatus, at the inner surface of the cell membrane by one of the HAS enzymes, where UDP-*N*-acetylglucosamine and UDP-glucuronic acid are added alternately to the reducing end of the HA chain being synthesized. The growing HA molecule then translocates extracellularly through the membrane. Studies have shown that HAS2 is responsible for the majority of HA synthesis and that HAS2 deletion in mice results in embryonic lethality due to severe cardiovascular defects [22–24].

**Figure 1:**
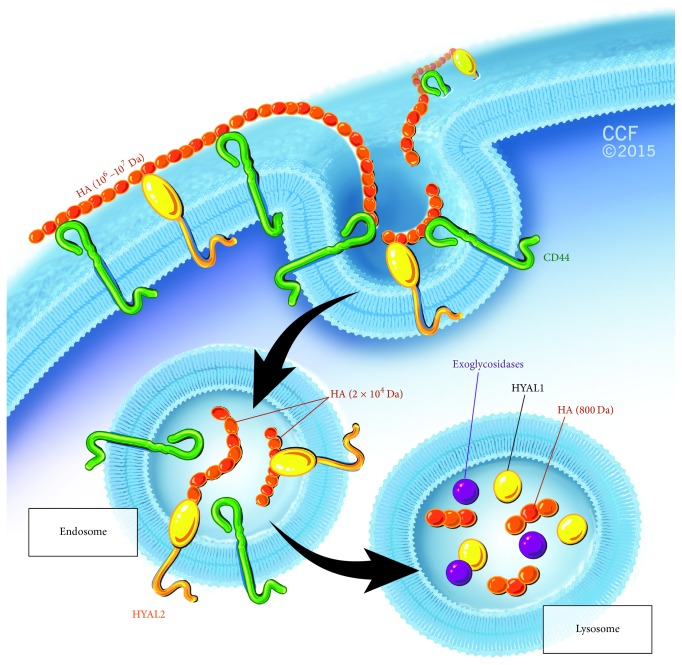
Schematic representation of a currently accepted model for cell-mediated HA degradation. Catabolism of HA starts at the cell surface. CD44 binds HA extracellularly and facilitates its degradation by HYAL2. The degradation products (now in the range of 20-kDa polymers) are internalized into endosomes and then transported into the lysosomes where they get further degraded by HYAL1 into tetrasaccharides.* N*-acetyl glucosaminidase and glucuronidase enzymes then further degrade HA into monosaccharides. “Reprinted with permission, Cleveland Clinic Center for Medical Art & Photography © 2015. All Rights Reserved”.

Under many conditions, HA exists in the body bound to one of its protein partners, such as CD44, versican, and aggrecan [25]. CD44 is ubiquitously expressed glycoprotein present on most mammalian cells and is considered the major cell surface receptor for HA. The interaction between CD44's cytoplasmic tail with many intracellular proteins, including kinases and cytoskeletal components, allows for HA to exert a wide range of different cell regulatory functions [26]. CD44 also has a role in HA catabolism by assisting HA-degrading enzymes. Catabolism of HA in humans occurs by endo-*β*-*N*-acetylhexosaminidase enzymes known as hyaluronidases (HYALs). Out of the six discovered HYAL enzymes, HYAL1 and HYAL2 are the only somatically active HA degrading enzymes in humans [27, 28] (Figure 1). HYAL1 was identified as an acid-active enzyme in serum in 1967 by De Salegui et al. and later confirmed to be a lysosomal enzyme [29, 30]. On the other hand, HYAL2, also an acid-active enzyme, is a glycosylphosphatidylinositol- (GPI-) anchored cell-surface protein [31]. The turnover of HA occurs rapidly in the body. HA is present normally in high amounts in multiple tissues and fluids of the body, including the joints, the eye vitreous, the umbilical cord, and amniotic fluid. High levels of HA are also present in proliferating tissues and tissues undergoing repair. As a result, the rapid catabolism of HA, through the activity of HYAL enzymes, represents a major mechanism by which HA levels are regulated in the body [32]. Studies have shown that HA degradation is dependent upon the classical HA binding receptor CD44 and involves mainly HYAL1 and HYAL2 [33, 34]. In addition to the enzymatic processes that cleave HA, HA can be degraded by oxidation reactions; particularly, reactive oxygen species (ROS) and free radicals are known to degrade HA polymers [35].

*(3) Hyaluronan and Inflammation.* Elevated levels of accumulated HA have been observed in many inflammatory diseases. For example, high levels of HA in joint tissues of patients with rheumatoid arthritis have been reported. Additionally, multiple studies have reported increased HA deposition in inflammatory diseases of the liver. Whereas the concentration of HA in the healthy liver is low, its concentration significantly increases in inflamed liver, leading to increased levels of serum HA. As a result, the level of circulating HA has been proposed as a biomarker for cirrhotic liver disease, for monitoring liver function, for assessing liver fibrosis, and for diagnosing chronic viral hepatitis C [36]. HA levels also increase in patients with inflammatory bowel disease (IBD), asthma, and idiopathic pulmonary arterial hypertension [37–39].

Accumulating evidence from studies published in the last 20 years has confirmed that the molecular weight of HA is critical in determining its biochemical and cellular roles. Numerous reports have shown that different sizes of HA exert a wide spectrum of functions [40, 41]. In tissues under normal conditions, HA is present in its high molecular weight (HMW-HA) form with an average size range of 1–10 × 10^6^ daltons. HMW-HA functions as a structural, hydrating polymer due to its hydrophilic properties. However, HMW-HA is also known to be anti-inflammatory. Large HA polymers have a role as molecules that indicate the integrity of tissues and control the cellular inflammatory responses [42]. For example, HMW-HA can protect from T-cell-mediated liver injury and bleomycin-mediated lung injury in mice and it can promote the suppressive effects of regulatory CD4^+^ CD25^+^ T cells [43–45]. Conversely, reports indicate that low molecular weight HA (LMW-HA) has proinflammatory effects. LMW-HA, or HA fragments that result from degradation of intact HMW-HA, has been shown to act as Damage Associated Molecular Patterns (molecules that can mediate and perpetuate an immune response in the absence of an infectious agent). Many reports have shown that fragmented HA is capable of signaling cellular responses through specific receptors, including CD44 and toll-like receptors (TLR) 2 and 4 [41, 42]. However, recent studies have shown that HA fragments can also induce another form of innate host defense responses, via TLR4, at the intestinal epithelium [46].

**Figure 2:**
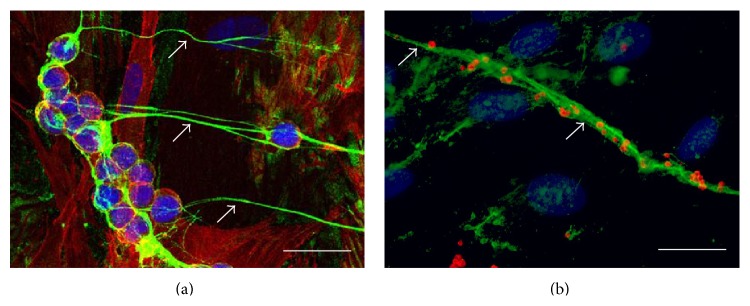
HA cables (green) on the surface of stimulated mucosal intestinal smooth muscle cells (M-SMCs) bind monocytes (round nuclei, red = CD44) (a) and platelets (red for CD42b) (b). Cultured M-SMCs were treated with polyI:C, a double-stranded RNA that mimics a viral infection, for 18 hours at 37°C. PolyI:C-stimulated M-SMCs were then coincubated with monocytes or platelets, methanol-fixed, and histochemically stained for HA (green and white arrows) and either CD44 (a) or CD42b (b) (scale bar = 25 *µ*m).

Under certain pathological conditions, HMW-HA can also become proinflammatory in the form of large leukocyte-adhesive, protein-decorated cable structures. During inflammation, inter-alpha-trypsin inhibitor (I*α*I), a serum protein, can leak into the extravascular spaces as a result of increased vascular permeability. The exposure of I*α*I to the extracellular matrix allows it to function as a heavy chain donor to HA. TNF-*α*-stimulated gene 6 (TSG-6), an enzyme and HA binding protein, facilitates the transfer of heavy chains 1 and 2 from I*α*I to HA to form leukocyte-adhesive HA cables. Importantly, TSG-6 expression increases in the inflamed tissues, which emphasizes the role HA cables play in inflammation [47]. Leukocyte-adhesive HA matrices have been reported in many inflammatory diseases, including intestinal tissues of IBD patients, lung tissues of asthmatic patients, lung tissues from idiopathic pulmonary hypertension patients, and synovial fluid of patients with arthritic disease [48, 49]. The production of HA cables can also be stimulated* in vitro* by inflammatory stimuli.  Figure 2 shows leukocytes (2A) and platelets (2B) bound specifically to HA cables produced by polyI:C-stimulated intestinal smooth muscle cells.

## 2. Hyaluronan and Fibrosis

**Figure 3:**
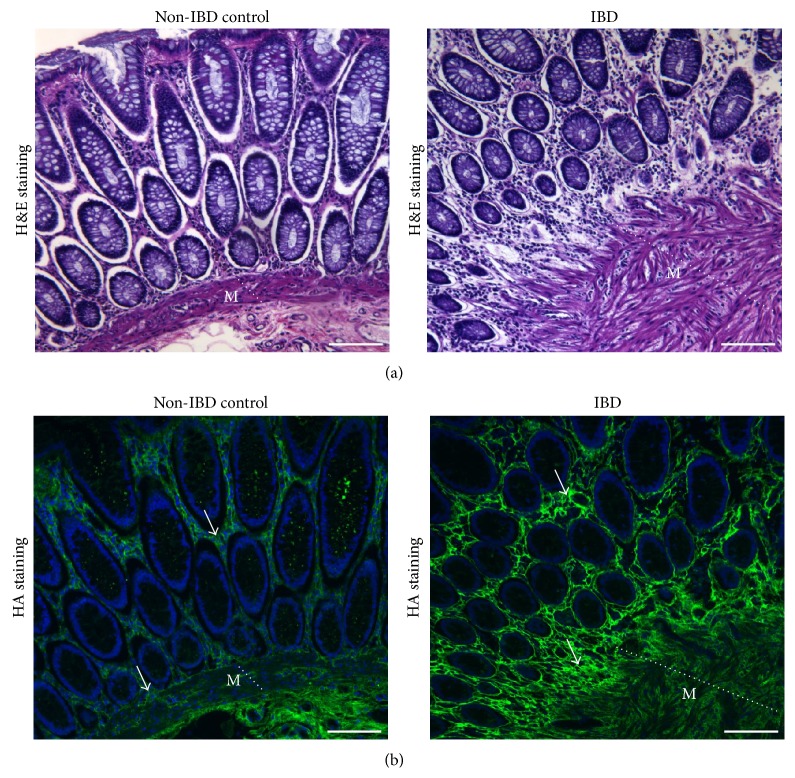
Colon tissue sections from a non-IBD and an IBD patient stained with hematoxylin & eosin (H&E) (a) or HA binding protein ((b), HA = green and DAPI = blue). The IBD colon shows symptoms of fibrosis, characterized by the expanded* muscularis mucosae* (dotted white line, M) compared to non-IBD control. Fluorescence histochemical staining shows elevated deposition of HA in both the epithelium and the* muscularis mucosae* as labeled by the white arrows. Scale bar (solid line) represents 100 *µ*m. IBD is a chronic inflammatory disease of unknown etiology. Development of fibrosis is a common and serious complication of IBD, one which requires surgical intervention to repair. It is thought that fibrosis in IBD stems from the chronic nature of inflammation signaling uncontrolled levels of wound healing.

HA plays an important role in fibrosis that has just recently become more appreciated. In the 1980s and 1990s, very few studies reported on the correlation between HA and fibrosis. However, a significantly increased number of publications have emerged in the last ten years suggesting a key role for HA in the fibrotic process, primarily in fibrotic lung and kidney. In 1989, Bjermer et al. compared HA levels in bronchoalveolar lavage (BAL) fluid from patients with idiopathic pulmonary fibrosis with those from healthy controls. The group reported that BAL HA levels in the tested patient population were five times higher than their healthy counterparts. However, serum HA levels in the idiopathic pulmonary fibrosis patients were comparable to those in the nondiseased population. Importantly, the increase in the amounts of HA in patients correlated significantly with the observed increase in BAL neutrophil and lymphocyte counts as well as the severity of the disease [50]. The same group later reported that HA levels, along with fibronectin levels, were significantly increased in the lung tissue, as well as in BAL fluid, during bleomycin-induced lung injury in rats. However, the accumulation of HA and fibronectin preceded the development of pulmonary fibrosis [51]. The data collectively suggest that HA may be playing an indirect role in promoting fibrosis: HA is known to bind and recruit immune cells and its accumulation before fibrosis suggests increased immune cell recruitment, as observed in patients with idiopathic pulmonary fibrosis. Immune cells, in turn, release a variety of inflammatory mediators and growth factors that are known to activate fibroblasts leading to fibrosis [3, 47–49]. Because HA is significantly increased in fibrotic tissues, multiple reports have suggested the use of HA levels as a biomarker for fibrosis, particularly for liver fibrosis [52, 53]. In addition, unpublished data from our lab shows increased deposition of HA in fibrotic intestines from IBD patients compared to non-IBD controls (Figure 3).

One of the first observations that TGF-*β* affects HA synthesis was in studies on limb development in the late 1980s. Synthesis of HA and pericellular coat formation in the mesoderm were stimulated by TGF-*β* [54]. In a concurrent study, when the effect of multiple growth factors on HA synthesis in cultured human foreskin fibroblasts was tested, not only did TGF-*β* stimulate HA production, but PDGF, epidermal growth factor, and basic fibroblast growth factor stimulated increased HA as well [55]. In multiple studies published in 1990, TGF-*β* was shown to promote HA production in cultured lung fibroblasts [56, 57]. The effect of TGF-*β* on HA synthesis in skin fibroblasts has also been reported. Whereas Westergren-Thorsson et al. found that TGF-*β* did not enhance HA production in skin fibroblasts [56], other studies have reported the opposite. For example, it was reported that TGF-*β* enhanced mRNA expression of HAS1 and HAS2 in cultured mouse skin fibroblasts [58]. Interestingly, Ellis and Schor reported that TGF-*β* inhibited HA synthesis by cultured skin fibroblasts when the cells were subconfluent, whereas it upregulated HA synthesis by confluent cells [59]. In human fibroblast-like synoviocytes, TGF-*β* was a strong stimulus for HAS1 transcription and HA synthase activity via a MAPK-dependent pathway, whereas it reduced HAS3 mRNA [60]. Strong evidence in the literature supports the hypothesis that TGF-*β*, which is a key inducer of fibrosis, also has an undeniable role in driving HA expression by fibroblasts.

One mechanism by which HA could be promoting fibrosis is through enhancing aberrant fibroblast motility. In addition to the production of HA, TGF-*β* also stimulates the expression of HA-mediated motility receptor (RHAMM), which, through interaction with HA, promotes cell locomotion. Samuel et al. showed that TGF-*β*, which is also a stimulator of motility, mediates the transcription and membrane expression of RHAMM along with HA production, resulting in an increase in motility response by cells [61]. The role of RHAMM-HA interaction in fibrogenesis was confirmed in later studies. Particularly, a peptide that specifically blocked HA-RHAMM binding but not HA-CD44 or HA-TLR binding was able to block fibroblast migration and alter wound repair in wild-type but not RHAMM-knockout mice. Additionally, the specific blockade of HA-RHAMM interaction caused a reduction in macrophage count and fibroblast number in excisional wounds in rats and blocked RHAMM-regulated focal adhesion kinase pathways in cultured fibroblasts [62]. The data suggest that RHAMM-HA interaction-mediated fibroblast migration contributes to inflammation and fibrogenesis and that targeting this interaction presents a novel approach to treating fibrosis.

Several reports suggested a role for HA in kidney fibrosis. Ito et al. reported that the treatment of cultured proximal tubular cells with HMW-HA and LMW-HA resulted, through activation of MAPK signaling cascade, in increased cell migration in scratch-wound assays. However, HMW-HA was a more potent stimulator of cell migration compared to LMW-HA. Interestingly, the effect of HA on cell migration was abrogated by blocking CD44. The group also tested the role of endogenous HA in cell migration and found that scratch-wounded cells produced significantly higher amounts of HA compared to control cells and that blocking CD44 or MAPK reduced cell migration [63]. One possibility is that the CD44 and RHAMM pathways that enhance cell migration are related and that HA needs to interact with both receptors in order to exert its cell-migratory effect that leads to fibrogenesis. In 2010, Han et al. suggested a role for HA and its receptors during interstitial fibrosis in chronic renal injury. The group reported that expression of HA, CD44, and lymphatic vessel endothelial hyaluronan receptor- (LYVE-) 1 increased in fibrotic tissue areas and that HA accumulation was accompanied by an increase in *α*-SMA [64]. More evidence supporting the role of HA in renal fibrosis comes from the studies of Kato et al. on basigin, which is a transmembrane protein known to regulate matrix metalloproteinase expression and enhance the production of HA in fibroblasts. The group found that basigin-deficient mice demonstrated significantly less fibrosis than wild-type animals after induced renal injury. Importantly, embryonic fibroblasts from basigin-deficient mice expressed lower levels of HAS2 than wild-type fibroblasts. In addition, TGF-*β* enhanced HAS2 expression, along with *α*-SMA mRNA, only in wild-type fibroblasts but not in basigin-deficient cells [65]. A recently published work by Colombaro et al. confirmed the possible role of HA in renal fibrosis. The researchers used deficient mice in one of the HA-degrading enzymes, HYAL1 or HYAL2, to demonstrate the effect of the increased accumulation of HA in the kidney following renal injury. Whereas HYAL1- and HYAL2-deficient mice suffered from intensified inflammation, wild-type mice demonstrated significant reduction in renal damage. In addition to increased HA accumulation, HYAL-deficient mice expressed increased levels of CD44, *α*-SMA, and collagen compared to wild-type mice 30 days after renal injury [66]. Data suggested that dynamic catabolism of HA, by the HYAL enzymes, is protective against renal injury by reducing the levels of accumulated HA that can contribute to kidney inflammation and fibrosis.

Steadman and Philips have extensively investigated the role of HA in fibrosis during the last 10 years. In an interesting study comparing the response to TGF-*β* stimulation between dermal and oral fibroblasts, the researchers found that TGF-*β*, through the activity of SMAD3, induced proliferation in dermal fibroblasts whereas it inhibited proliferation in oral fibroblasts. Importantly, levels of HA released by dermal fibroblasts were significantly higher than those released by oral fibroblasts and blocking HA synthesis in dermal fibroblasts inhibited the proliferative function of TGF-*β* [67]. The study is important because it demonstrates that the effect of TGF-*β* depends on the levels of HA produced in fibroblasts; after injury, HA appears to be a key factor that contributes to TGF-*β*-induced scar formation, whereas in oral fibroblasts, which are known to heal without scarring and fibrosis, HA levels are low. In a follow-up study, the group showed that TGF-*β*-dependent fibroblast proliferation depended on the expression of CD44 and that CD44-EGFR interaction is required for the proliferative effect of TGF-*β* via MAPK/ERK pathway. Interestingly, the researchers were able to induce TGF-*β*-dependent proliferation in oral fibroblasts by overexpressing HAS2, confirming the role of accumulated HA in fibrosis [68]. In other studies, the group showed that TGF-*β*-dependent fibroblast-to-myofibroblast transformation is also dependent upon HA/CD44/EGFR involvement [69]. In 2015, Midgley et al. reported on the role of HA catabolism in the fibrotic process, particularly in myofibroblast differentiation. The group showed that Bone Morphogenetic Protein 7, which is a cytokine known to have antifibrotic effect, prevented TGF-*β*-dependent lung myofibroblast differentiation by promoting cell-surface HA internalization and degradation by HYAL2 and CD44 variant isoform CD44V7/8 [70]. The reports published by Steadman and Philips suggest collectively that targeting proteins involved in HA production or degradation represents a unique approach to the prevention and probably the reversal of fibrosis. More evidence confirming the central role of HA and CD44 in pulmonary fibrosis, particularly* in vivo*, was reported by Li et al. The researchers overexpressed HAS2 specifically in the myofibroblasts of mice and found that these animals demonstrated severe fibrosis and higher mortality than wild-type mice after bleomycin-induced lung injury. In an assay that evaluated the invasiveness of fibroblasts in a composite matrix with basement membrane constituents, the group found that fibroblasts isolated from bleomycin-treated mice invaded matrix more readily than control fibroblasts and that this invasion was dependent upon the expression of HAS2 and CD44. Importantly, the study showed that development of lung fibrosis* in vivo* was also dependent on HAS2 and CD44 expression [71].

Although the majority of reports in the literature suggest that HA promotes fibrogenesis and myofibroblast differentiation, the findings of a few researchers suggest the opposite. For example, Evanko et al. reported that HA controls fibrosis by binding to fibrillar matrix components. The group found that TGF-*β* treatment of lung fibroblasts resulted in the colocalization of HA and fibronectin in the ECM of the formed myofibroblasts. Importantly, inhibition of HA synthesis or disruption of ECM-HA resulted, surprisingly, in increased fibronectin and collagen deposition as well as increased *α*-SMA expression and myofibroblast phenotype enhancement [72]. It is unclear why this discrepancy exists; however, it suggests that HA's role in myofibroblasts is complex and more research is needed to better understand the role of HA in fibrosis. In 2012, Li et al. took a totally novel approach to study the role of HA in fibrosis, particularly in synovial fibrosis* in vivo*. Using an osteoarthritis mouse model, the researchers tested the effect of HA injection in abrogating fibrosis and the tissue changes that result from TGF-*β* treatment. Interestingly, the group found that HA treatment protected against TGF-*β*-induced fibrosis in the wild-type animals. However, HA treatment had no protecting effect on CD44-knockout mice, suggesting a role for HA-CD44 binding in exerting the protective effects of HA [73].

It is unknown whether HMW-HA or LMW-HA has the most potent effect in driving fibrosis or whether size matters at all in the fibrotic process. Most studies on the role of HA in fibrosis were performed assuming that HA is in its native form, which is generally its HMW-HA form. One of the exceptions is the work mentioned above from Turley's laboratory on the effect of RHAMM on fibrogenesis [62]. The researchers found that LMW-HA had higher binding affinity to RHAMM than HMW-HA and predicted that HA fragments are essential promoters of fibrosis, which is also partially based on the fact that LMW-HA is well-known to be proinflammatory [42]. A possible mechanism by which LMW-HA promotes fibrosis is through driving aberrant wound healing. In one study, Tolg et al. measured the effect of HA fragments on promoting wound healing and showed that LMW-HA stimulated dermal fibroblast migration and closure of excisional wounds. Furthermore, the group found that HA fragments increased the accumulation of TGF-*β*1 and the infiltration of macrophages in the wound [74]. Work by other groups showed that LMW-HA stimulated increased expression and production of TGF-*β*3, collagen, and tissue inhibitor of matrix metalloproteinase in dermal fibroblasts and endothelial cells [75, 76]. The promotion of wound healing, production of TGF-*β*, and deposition of collagen by LMW-HA suggest a different mechanism by which HA can promote fibrosis, one that possibly involves inflammation. Because levels of HA in fibrotic conditions are reported to be high, more HA fragments could be created, leading to uncontrolled wound healing and, subsequently, to fibrosis.

## 3. Conclusion

The presented body of evidence suggests that HA is important in fibrotic repair response and investigating fibrosis in the light of HA regulation is essential. Furthermore, because the molecular weight of HA generally determines its function, investigating what role different HA sizes have in fibrogenesis might open new doors to understanding the pathogenic process that leads to fibrosis. Our previous knowledge about HA in fibrosis was limited to the earlier publications that mostly reported on a correlation between increased HA levels and fibrosis, which suggested the use of HA as a biomarker for diagnosing fibrosis. However, recent work, particularly from labs of Steadman, Philips, Turley, and Noble, clearly showed that HA, in fact, is not just a passive player or an outcome of fibrosis but rather a driving factor that is required for TGF-*β* to exert its profibrotic effects. HA contributes to fibrosis by mediating fibroblast motility, fibroblast proliferation, and fibroblast-to-myofibroblast differentiation. In addition, reports have linked HA receptors, particularly CD44 and RHAMM, as well as HA synthases and degrading enzymes to fibrosis. This suggests that HA and its regulation pathways potentially represent novel targets for antifibrotic therapies.

## References

[1] Halper J., Kjaer M. (2014). Basic components of connective tissues and extracellular matrix: elastin, fibrillin, fibulins, fibrinogen, fibronectin, laminin, tenascins and thrombospondins. *Advances in Experimental Medicine and Biology*.

[2] Cox T. R., Erler J. T. (2011). Remodeling and homeostasis of the extracellular matrix: implications for fibrotic diseases and cancer. *Disease Models & Mechanisms*.

[3] Rieder F., Fiocchi C. (2009). Intestinal fibrosis in IBD—a dynamic, multifactorial process. *Nature Reviews Gastroenterology and Hepatology*.

[4] Bonnans C., Chou J., Werb Z. (2014). Remodelling the extracellular matrix in development and disease. *Nature Reviews Molecular Cell Biology*.

[5] Hinz B., Phan S. H., Thannickal V. J., Galli A., Bochaton-Piallat M.-L., Gabbiani G. (2007). The myofibroblast: one function, multiple origins. *The American Journal of Pathology*.

[6] Baum J., Duffy H. S. (2011). Fibroblasts and myofibroblasts: what are we talking about?. *Journal of Cardiovascular Pharmacology*.

[7] Rajkumar V. S., Shiwen X., Bostrom M. (2006). Platelet-derived growth factor-*β* receptor activation is essential for fibroblast and pericyte recruitment during cutaneous wound healing. *American Journal of Pathology*.

[8] Sullivan D. E., Ferris M., Nguyen H., Abboud E., Brody A. R. (2009). TNF-*α* induces TGF-*β*1 expression in lung fibroblasts at the transcriptional level via AP-1 activation. *Journal of Cellular and Molecular Medicine*.

[9] Kolodsick J. E., Toews G. B., Jakubzick C. (2004). Protection from fluorescein isothiocyanate-induced fibrosis in IL-13-deficient, but not IL-4-deficient, mice results from impaired collagen synthesis by fibroblasts. *The Journal of Immunology*.

[10] Mchedlidze T., Waldner M., Zopf S. (2013). Interleukin-33-dependent innate lymphoid cells mediate hepatic fibrosis. *Immunity*.

[11] Leask A., Abraham D. J. (2004). TGF-*β* signaling and the fibrotic response. *The FASEB Journal*.

[12] Verrecchia F., Chu M.-L., Mauviel A. (2001). Identification of novel TGF-*β*/Smad gene targets in dermal fibroblasts using a combined cDNA microarray/promoter transactivation approach. *The Journal of Biological Chemistry*.

[13] Serini G., Bochaton-Piallat M.-L., Ropraz P. (1998). The fibronectin domain ED-A is crucial for myofibroblastic phenotype induction by transforming growth factor-*β*1. *Journal of Cell Biology*.

[14] Bhattacharyya S., Tamaki Z., Wang W. (2014). FibronectinEDA promotes chronic cutaneous fibrosis through toll-like receptor signaling. *Science Translational Medicine*.

[15] Lipson K. E., Wong C., Teng Y., Spong S. (2012). CTGF is a central mediator of tissue remodeling and fibrosis and its inhibition can reverse the process of fibrosis. *Fibrogenesis & Tissue Repair*.

[16] Bensadoun E. S., Burke A. K., Hogg J. C., Roberts C. R. (1996). Proteoglycan deposition in pulmonary fibrosis. *The American Journal of Respiratory and Critical Care Medicine*.

[17] Westergren-Thorsson G., Hernnäs J., Särnstrand B., Oldberg Å., Heinegård D., Malmström A. (1993). Altered expression of small proteoglycans, collagen, and transforming growth factor-*β*1 in developing bleomycin-induced pulmonary fibrosis in rats. *The Journal of Clinical Investigation*.

[18] Venkatesan N., Ebihara T., Roughley P. J., Ludwig M. S. (2000). Alterations in large and small proteoglycans in bleomycin-induced pulmonary fibrosis in rats. *American Journal of Respiratory and Critical Care Medicine*.

[19] Ebihara T., Venkatesan N., Tanaka R., Ludwig M. S. (2000). Changes in extracellular matrix and tissue viscoelasticity in bleomycin-induced lung fibrosis: temporal aspects. *American Journal of Respiratory and Critical Care Medicine*.

[20] Venkatesan N., Tsuchiya K., Kolb M. (2014). Glycosyltransferases and glycosaminoglycans in bleomycin and transforming growth factor-*β*1-induced pulmonary fibrosis. *American Journal of Respiratory Cell and Molecular Biology*.

[21] Toole B. P. (2004). Hyaluronan: from extracellular glue to pericellular cue. *Nature Reviews Cancer*.

[22] Itano N., Kimata K. (2002). Mammalian hyaluronan synthases. *IUBMB Life*.

[23] Vigetti D., Karousou E., Viola M., Deleonibus S., de Luca G., Passi A. (2014). Hyaluronan: biosynthesis and signaling. *Biochimica et Biophysica Acta*.

[24] Wang Y., Lauer M. E., Anand S., Mack J. A., Maytin E. V. (2014). Hyaluronan synthase 2 protects skin fibroblasts against apoptosis induced by environmental stress. *The Journal of Biological Chemistry*.

[25] Kohda D., Morton C. J., Parkar A. A. (1996). Solution structure of the link module: a hyaluronan-binding domain involved in extracellular matrix stability and cell migration. *Cell*.

[26] Naor D., Sionov R. V., Ish-Shalom D. (1997). CD44: structure, function, and association with the malignant process. *Advances in Cancer Research*.

[27] Csoka A. B., Frost G. I., Stern R. (2001). The six hyaluronidase-like genes in the human and mouse genomes. *Matrix Biology*.

[28] Kaneiwa T., Mizumoto S., Sugahara K., Yamada S. (2010). Identification of human hyaluronidase-4 as a novel chondroitin sulfate hydrolase that preferentially cleaves the galactosaminidic linkage in the trisulfated tetrasaccharide sequence. *Glycobiology*.

[29] De Salegui M., Pigman W. (1967). The existence of an acid-active hyaluronidase in serum. *Archives of Biochemistry and Biophysics*.

[30] Triggs-Raine B., Salo T. J., Zhang H., Wicklow B. A., Natowicz M. R. (1999). Mutations in HYAL1, a member of a tandemly distributed multigene family encoding disparate hyaluronidase activities, cause a newly described lysosomal disorder, mucopolysaccharidosis IX. *Proceedings of the National Academy of Sciences of the United States of America*.

[31] Albeiroti S., Ayasoufi K., Hill D. R., Shen B., de la Motte C. A. (2015). Platelet hyaluronidase-2: an enzyme that translocates to the surface upon activation to function in extracellular matrix degradation. *Blood*.

[32] Stern R., Jedrzejas M. J. (2006). Hyaluronidases: their genomics, structures, and mechanisms of action. *Chemical Reviews*.

[33] Bourguignon L. Y. W., Singleton P. A., Diedrich F., Stern R., Gilad E. (2004). CD44 interaction with Na^+^-H^+^ exchanger (NHE1) creates acidic microenvironments leading to hyaluronidase-2 and cathepsin B activation and breast tumor cell invasion. *Journal of Biological Chemistry*.

[34] Harada H., Takahashi M. (2007). CD44-dependent intracellular and extracellular catabolism of hyaluronic acid by hyaluronidase-1 and -2. *The Journal of Biological Chemistry*.

[35] Monzon M. E., Fregien N., Schmid N. (2010). Reactive oxygen species and hyaluronidase 2 regulate airway epithelial hyaluronan fragmentation. *The Journal of Biological Chemistry*.

[36] Guechot J., Loria A., Serfaty L., Giral P., Giboudeau J., Poupon R. (1995). Serum hyaluronan as a marker of liver fibrosis in chronic viral hepatitis C: effect of *α*-interferon therapy. *Journal of Hepatology*.

[37] Liang J., Jiang D., Jung Y. (2011). Role of hyaluronan and hyaluronan-binding proteins in human asthma. *Journal of Allergy and Clinical Immunology*.

[38] Kessler S., Rho H., West G., Fiocchi C., Drazba J., de la Motte C. (2008). Hyaluronan (HA) deposition precedes and promotes leukocyte recruitment in intestinal inflammation. *Clinical and Translational Science*.

[39] Aytekin M., Comhair S. A. A., de la Motte C. (2008). High levels of hyaluronan in idiopathic pulmonary arterial hypertension. *The American Journal of Physiology: Lung Cellular and Molecular Physiology*.

[40] Campo G. M., Avenoso A., D'Ascola A. (2012). Hyaluronan differently modulates TLR-4 and the inflammatory response in mouse chondrocytes. *BioFactors*.

[41] Stern R., Asari A. A., Sugahara K. N. (2006). Hyaluronan fragments: an information-rich system. *European Journal of Cell Biology*.

[42] Petrey A. C., de la Motte C. A. (2014). Hyaluronan, a crucial regulator of inflammation. *Frontiers in Immunology*.

[43] Nakamura K., Yokohama S., Yoneda M. (2004). High, but not low, molecular weight hyaluronan prevents T-cell-mediated liver injury by reducing proinflammatory cytokines in mice. *Journal of Gastroenterology*.

[44] Teder P., Vandivier R. W., Jiang D. (2002). Resolution of lung inflammation by CD44. *Science*.

[45] Bollyky P. L., Lord J. D., Masewicz S. A. (2007). Cutting edge: high molecular weight hyaluronan promotes the suppressive effects of CD4^+^CD25^+^ regulatory T cells. *The Journal of Immunology*.

[46] Hill D. R., Kessler S. P., Rho H. K., Cowman M. K., de la Motte C. A. (2012). Specific-sized hyaluronan fragments promote expression of human *β*-defensin 2 in intestinal epithelium. *The Journal of Biological Chemistry*.

[47] Lauer M. E., Glant T. T., Mikecz K. (2013). Irreversible heavy chain transfer to hyaluronan oligosaccharides by tumor necrosis factor-stimulated gene-6. *The Journal of Biological Chemistry*.

[48] Zhao M., Yoneda M., Ohashi Y. (1995). Evidence for the covalent binding of SHAP, heavy chains of inter-*α*-trypsin inhibitor, to hyaluronan. *The Journal of Biological Chemistry*.

[49] de la Motte C. A., Hascall V. C., Drazba J., Bandyopadhyay S. K., Strong S. A. (2003). Mononuclear leukocytes bind to specific hyaluronan structures on colon mucosal smooth muscle cells treated with polyinosinic acid:polycytidylic acid: inter-*α*-trypsin inhibitor is crucial to structure and function. *The American Journal of Pathology*.

[50] Bjermer L., Lundgren R., Hallgren R. (1989). Hyaluronan and type III procollagen peptide concentrations in bronchoalveolar lavage fluid in idiopathic pulmonary fibrosis. *Thorax*.

[51] Hernnas J., Nettelbladt O., Bjermer L., Särnstrand B., Malmström A., Hällgren R. (1992). Alveolar accumulation of fibronectin and hyaluronan precedes bleomyin-induced pulmonary fibrosis in the rat. *European Respiratory Journal*.

[52] Halfon P., Bourlière M., Pénaranda G. (2005). Accuracy of hyaluronic acid level for predicting liver fibrosis stages in patients with hepatitis C virus. *Comparative Hepatology*.

[53] Guéchot J., Loria A., Serfaty L., Giral P., Giboudeau J., Poupon R. (1995). Serum hyaluronan as a marker of liver fibrosis in chronic viral hepatitis C: effect of *α*-interferon therapy. *Journal of Hepatology*.

[54] Toole B. P., Munaim S. I., Welles S., Knudson C. B. (1989). Hyaluronate-cell interactions and growth factor regulation of hyaluronate synthesis during limb development. *Ciba Foundation symposium*.

[55] Heldin P., Laurent T. C., Heldin C.-H. (1989). Effect of growth factors on hyaluronan synthesis in cultured human fibroblasts. *Biochemical Journal*.

[56] Westergren-Thorsson G., Särnstrand B., Fransson L.-A., Malmström A. (1990). TGF-*β* enhances the production of hyaluronan in human lung but not in skin fibroblasts. *Experimental Cell Research*.

[57] Dubaybo B. A., Thet L. A. (1990). Effect of transforming growth factor beta on synthesis of glycosaminoglycans by human lung fibroblasts. *Experimental Lung Research*.

[58] Sugiyama Y., Shimada A., Sayo T., Sakai S., Inoue S. (1998). Putative hyaluronan synthase mRNA are expressed in mouse skin and TGF-*β* upregulates their expression in cultured human skin cells. *Journal of Investigative Dermatology*.

[59] Ellis I. R., Schor S. L. (1996). Differential effects of TGF-*β*1 on hyaluronan synthesis by fetal and adult skin fibroblasts: implications for cell migration and wound healing. *Experimental Cell Research*.

[60] Stuhlmeier K. M., Pollaschek C. (2004). Differential effect of transforming growth factor beta (TGF-beta) on the genes encoding hyaluronan synthases and utilization of the p38 MAPK pathway in TGF-beta-induced hyaluronan synthase 1 activation. *Journal of Biological Chemistry*.

[61] Samuel S. K., Hurta R. A. R., Spearman M. A., Wright J. A., Turley E. A., Greenberg A. H. (1993). TGF-*β*1 stimulation of cell locomotion utilizes the hyaluronan receptor RHAMM and hyaluronan. *The Journal of Cell Biology*.

[62] Tolg C., Hamilton S. R., Zalinska E. (2012). A RHAMM mimetic peptide blocks hyaluronan signaling and reduces inflammation and fibrogenesis in excisional skin wounds. *American Journal of Pathology*.

[63] Ito T., Williams J. D., Al-Assaf S., Phillips G. O., Phillips A. O. (2004). Hyaluronan and proximal tubular cell migration. *Kidney International*.

[64] Han D. H., Song H. K., Lee S. Y. (2010). Upregulation of hyaluronan and its binding receptors in an experimental model of chronic cyclosporine nephropathy. *Nephrology*.

[65] Kato N., Kosugi T., Sato W. (2011). Basigin/CD147 promotes renal fibrosis after unilateral ureteral obstruction. *American Journal of Pathology*.

[66] Colombaro V., Jadot I., Declèves A. E. (2015). Lack of hyaluronidases exacerbates renal post-ischemic injury, inflammation, and fibrosis. *Kidney International*.

[67] Meran S., Thomas D. W., Stephens P. (2008). Hyaluronan facilitates transforming growth factor-*β*1-mediated fibroblast proliferation. *The Journal of Biological Chemistry*.

[68] Meran S., Luo D. D., Simpson R. (2011). Hyaluronan facilitates transforming growth factor-*β*1-dependent proliferation via CD44 and epidermal growth factor receptor interaction. *The Journal of Biological Chemistry*.

[69] Midgley A. C., Rogers M., Hallett M. B. (2013). Transforming growth factor-*β*1 (TGF-*β*1)-stimulated fibroblast to myofibroblast differentiation is mediated by hyaluronan (HA)-facilitated epidermal growth factor receptor (EGFR) and CD44 co-localization in lipid rafts. *Journal of Biological Chemistry*.

[70] Midgley A. C., Duggal L., Jenkins R. (2015). Hyaluronan regulates bone morphogenetic protein-7-dependent prevention and reversal of myofibroblast phenotype. *Journal of Biological Chemistry*.

[71] Li Y., Jiang D., Liang J. (2011). Severe lung fibrosis requires an invasive fibroblast phenotype regulated by hyaluronan and CD44. *Journal of Experimental Medicine*.

[72] Evanko S. P., Potter-Perigo S., Petty L. J., Workman G. A., Wight T. N. (2015). Hyaluronan controls the deposition of fibronectin and collagen and modulates TGF-*β*1 induction of lung myofibroblasts. *Matrix Biology*.

[73] Li J., Gorski D. J., Anemaet W. (2012). Hyaluronan injection in murine osteoarthritis prevents TGFbeta 1-induced synovial neovascularization and fibrosis and maintains articular cartilage integrity by a CD44-dependent mechanism. *Arthritis Research and Therapy*.

[74] Tolg C., Telmer P., Turley E., Mukhopadhyay P. (2014). Specific sizes of hyaluronan oligosaccharides stimulate fibroblast migration and excisional wound repair. *PLoS ONE*.

[75] David-Raoudi M., Tranchepain F., Deschrevel B. (2008). Differential effects of hyaluronan and its fragments on fibroblasts: relation to wound healing. *Wound Repair and Regeneration*.

[76] Rooney P., Wang M., Kumar P., Kumar S. (1993). Angiogenic oligosaccharides of hyaluronan enhance the production of collagens by endothelial cells. *The Journal of Cell Science*.

